# Bikeability and methodological issues using the active commuting route environment scale (ACRES) in a metropolitan setting

**DOI:** 10.1186/1471-2288-11-6

**Published:** 2011-01-17

**Authors:** Lina Wahlgren, Peter Schantz

**Affiliations:** 1 The Research Unit for Movement, Health and Environment, The Åstrand Laboratory, GIH - The Swedish School of Sport and Health Sciences, SE-114 86 Stockholm, Sweden; 2 School of Health and Medical Sciences, Örebro University, SE-701 82 Örebro, Sweden; 3 Department of Health Sciences, Mid Sweden University, SE-831 25 Östersund, Sweden

## Abstract

**Background:**

Route environments can positively influence people's active commuting and thereby contribute to public health. The Active Commuting Route Environment Scale (ACRES) was developed to study active commuters' perceptions of their route environments. However, bicycle commuters represent a small portion of the population in many cities and thus are difficult to study using population-based material. Therefore, the aim of this study is to expand the state of knowledge concerning the criterion-related validity of the ACRES and the representativity using an advertisement-recruited sample. Furthermore, by comparing commuting route environment profiles of inner urban and suburban areas, we provide a novel basis for understanding the relationship between environment and bikeability.

**Methods:**

Bicycle commuters from Greater Stockholm, Sweden, advertisement- (n = 1379) and street-recruited (n = 93), responded to the ACRES. Traffic planning and environmental experts from the Municipality of Stockholm (n = 24) responded to a modified version of the ACRES. The criterion-related validity assessments were based on whether or not differences between the inner urban and the suburban route environments, as indicated by the experts and by four existing objective measurements, were reflected by differences in perceptions of these environments. Comparisons of ratings between advertisement- and street-recruited participants were used for the assessments of representativity. Finally, ratings of inner urban and suburban route environments were used to evaluate commuting route environment profiles.

**Results:**

Differences in ratings of the inner urban and suburban route environments by the advertisement-recruited participants were in accord with the existing objective measurements and corresponded reasonably well with those of the experts. Overall, there was a reasonably good correspondence between the advertisement- and street-recruited participants' ratings. Distinct differences in commuting route environment profiles were noted between the inner urban and suburban areas. Suburban route environments were rated as safer and more stimulating for bicycle-commuting than the inner urban ones. In general, the findings applied to both men and women.

**Conclusions:**

The overall results show: considerable criterion-related validity of the ACRES; ratings of advertisement-recruited participants mirroring those of street-recruited participants; and a higher degree of bikeability in the suburban commuting route environments than in the inner urban ones.

## Background

Increasing the population-wide level of physical activity is a major public health concern [e.g. [1]]. Active commuting could constitute an important potential in this respect [for a review, see [2]], and the route environment could be a potentially important factor in influencing people's active commuting behaviours. Both objective aspects and people's perceptions of the environments are likely to influence behaviours [cf. [3]]. Thus, understanding the possible relationships between active commuting and the route environments is important.

Review articles on the relation between the physical environment and physical activity in general indicate that mixed land use, residential density, street connectivity and physical infrastructure, such as pavements, are associated with levels of physical activity [for a review of reviews, see [4]]. These findings are mainly based on three different measures of the environment: (1) perceived measures, based on self-report questionnaires; (2) observational measures, based on audit tools; and (3) objective measures, based on Geographical Information Systems (GIS) [for a review, see [5]]. The majority of these measures, and particularly the self-report questionnaires, deal primarily with the local neighbourhood environment, often defined as the area within a 10 to 15-minute walk from your home or similar definitions [e.g. [6]]. Active commuting, however, often involves an extended environment compared to the local neighbourhood [7,8]. We have therefore developed a scale called the Active Commuting Route Environment Scale (ACRES), which is based on a complete spatial matching between the environment and the physical activity variable [9].

The ACRES was developed for the assessment of bicyclists' and pedestrians' perceptions of different factors in their commuting route environment. It can be used for different purposes. One is to enable evaluations of how different settings are rated in terms of traffic, physical and social environmental variables, as well as in terms of perceptions of traffic safety and whether a route environment stimulates or hinders walking or bicycle commuting. Not least since traffic unsafety appears to be a major hinder to active transportation [cf. [10]], understanding environmental correlates to this perception is important in a public health perspective.

We have previously assessed the reliability and the validity of the ACRES for bicyclists, using a street-recruited sample of bicycle commuters, and found that it was characterized by considerable criterion-related validity and reasonable test-retest reproducibility [9]. This assessment of the criterion-related validity was based on a comparison between inner urban and suburban route environments. Ratings by the bicycle commuters were compared with ratings by an expert panel, and four of the items were also compared with existing objective measures.

Since the psychometric evaluation of the ACRES has not been repeated, and it is important to establish a stable basis before using the scale more broadly, we considered it to be useful to study the criterion-related validity on other and larger groups of active commuters, while also enabling separate analyses of men and women. This is the first aim of this study. The second aim deals with the fact that active commuters, particularly bicycle commuters, often represent a small group within the general population. It is therefore challenging to study a larger and representative group within this subpopulation. In our previous study, we recruited participants in the street, while they were walking or cycling. We consider this recruitment strategy a realistic approach reaching out to a representative group of active commuters. This is, however, a time-consuming method, and a wide geographical coverage, not the least in a metropolitan setting, is still difficult to obtain. As an alternative method, we have recruited participants by advertisements in morning newspapers. As an index of comparability of the sampling methods, we considered it important to compare the ratings of the ACRES between advertisement- and street-recruited participants. The third aim is to study the commuting route environment profiles of the inner urban and suburban areas. This provides both evidence regarding the criterion-related validity and a novel basis for furthering the understanding of different environments in relation to two important aspects of bikeability: namely, the perception of traffic safety and whether the overall route environment stimulates or hinders active commuting and how this can relate to different aspects of the physical, traffic and social environments. This assessment was based on the advertisement-recruited participants, thereby enabling separate analyses of men and women, and also a separation of those who bicycle-commuted in both inner urban and suburban areas from those who bicycle-commuted in only an inner urban or a suburban area. Unless indicated in the following, we had no directed hypotheses regarding particular rating response patterns for any of the subgroups, sexes and issues studied.

## Methods

### Study design

The ACRES addresses the inner urban and suburban route environments separately. In a previous study, we therefore could use expected differences between the two environments for a criterion-related validity assessment of the ACRES [9]. The 'known group difference method' [cf. [11]] served as a model. We then used a street-recruited group of active bicycle commuters as well as existing objective measures and an expert panel for the assessments. Commuters who cycled in both the inner urban and suburban areas were used. The experts provided data on both route environments. In this study we use the same design, but instead a group of active bicycle commuters were recruited by advertisements in morning newspapers. Consequently, for the first part of the criterion-related validity assessment in this study, we used participants who bicycle-commuted in both inner urban and suburban areas. The directions of differences between the inner urban and the suburban route environments rated by the bicycle commuters were compared with the directions of differences of four existing objective measures. Thereafter, the sizes of differences in the ratings of inner urban and suburban route environments, separated for men and women, were compared with the experts' ratings.

We were concerned about the representativity of the advertisement-recruited participants. Therefore, we compared the ratings of the inner urban and suburban route environments between the advertisement- and street-recruited participants. From both groups, participants who provided data in both inner and suburban route environments were used. The ratings of inner urban and suburban route environments by the two groups were used for comparisons of both absolute levels, distributions of values and directions and sizes of differences between the inner urban and suburban environments. Data from the street-recruited sample were also used as part of the assessment of criterion-related validity.

Finally, we evaluated the commuting route environment profiles of the inner urban and suburban areas. This assessment was based on a larger group of the advertisement-recruited participants, separated into men and women, divided into the following subgroups: (1) those who bicycle-commuted in both inner urban and suburban areas (hereafter called *Both I&S*); (2) those who bicycle-commuted in only an inner urban area (hereafter called *Only I*); and (3) those who bicycle-commuted in only a suburban area (hereafter called *Only S*). The ratings of the inner urban and the suburban route environments by *Both I&S *were compared. Furthermore, the ratings of the inner urban route environment by *Only I *and the ratings of the suburban route environment by *Only S *were compared. These comparisons were used in part for the assessment of criterion-related validity. The different commuting route environment profiles provided a basis for furthering understanding of different environments in relation to important aspects of bikeability. Finally, the ratings of the inner urban route environments were compared between *Both I&S *and *Only I*, and the ratings of the suburban route environments were compared between *Both I&S *and *Only S*. This separation into subgroups enabled an expanded understanding of the validity of the ACRES in terms of whether or not bicycling and ratings of route environments in two different areas, as compared to only one area, would affect the rating levels.

Each of these three parts will be presented as distinct entities in the Results, whereas in the Discussion, data from the three different parts will be combined to address the issue of criterion-related validity.

The Ethics Committee of the Karolinska Institute approved the study. The participants gave their informed consent.

### Advertisement-recruited participants

The participants were recruited with the aim of attaining a reasonable reflection of the adult active commuters in the inner urban and suburban areas of Greater Stockholm, Sweden, during the recruitment period. Active commuters represent a small group within the general population and, therefore, it was not possible, in practical terms, to recruit a sufficient number of participants from a random population sample. We therefore recruited participants by advertising in two large morning newspapers (Dagens Nyheter and Svenska Dagbladet) in Stockholm towards the end of May and early June 2004. Inclusion criteria included: (a) being at least 20 years old; (b) living in Stockholm County, excluding the municipality of Norrtälje; and (c) walking and/or cycling the whole way to one's place of work or study at least once a year. In the invitation to participate, we emphasized that people with short commuting distances were also welcome to participate. The reason for including people with less frequent active commuting behaviours, as well as with short route distances, was to include a wide range of commuting behaviours.

The advertisement resulted in 2148 people volunteering to take part. We posted a first questionnaire, named the Physically Active Commuting in Greater Stockholm Questionnaire (PACS Q1), to the participants in September 2004. The response frequency was 94% (n = 2010). During the peak bicycle-commuting period of the year, in May 2005, a second questionnaire, the PACS Q2, was sent to 1978 participants. (For descriptions of the PACS Q1 and Q2, see below.) The response frequency was 92% (n = 1819). Both questionnaires were sent home to each participant together with a prepaid return envelop. A maximum of three reminders were sent out. No incentives were provided for the participants. We excluded some participants because they did not meet the inclusion criteria or did not wish to participate in the second part of the study. The participants were bicyclists, pedestrians or dual-mode performers, i.e. individuals who sometimes walk and sometimes cycle. They commuted in the inner urban, suburban - rural or both inner and suburban - rural areas of Greater Stockholm, Sweden. The suburban - rural areas are hereafter referred to as suburban areas, unless stated otherwise. We have only used data on bicycle-commuting in this study. After cleansing and editing the data, 1379 participants (women, n = 898, 64%) were included in the analyses. The participants yielded data in the following subgroups: *Both I&S *(n = 555: women, n = 302, 54%); *Only I *(n = 272: women, n = 197, 72%); and *Only S *(n = 552: women, n = 399, 72%). For further descriptive characteristics of the participants, see Table 1.

**Table 1:** Descriptive characteristics of advertisement-recruited and street-recruited participants

	Advertisement-recruited participants						Street-recruited participants	
			
	Both I&S*		Only I*		Only S*			
				
Characteristic	Women (n = 299-302)	Men (n = 249-253)	Women (n = 193-197)	Men (n = 73-75)	Women (n = 391-399)	Men (n = 151-153)	Women (n = 32-33)	Men (n = 59-60)
Age in years, mean ± SD	46.9 ± 9.9	48.2 ± 10.6	47.5 ± 11.3	46.2 ± 11.9	49.4 ± 10.0	49.4 ± 10.6	42.0 ± 9.0	45.9 ± 10.7
Weight in kg, mean ± SD	64.5 ± 8.2	78.9 ± 10.6	63.8 ± 7.9	77.6 ± 8.3	65.0 ± 9.2	78.4 ± 9.4	61.8 ± 7.3	78.0 ± 10.0
Height in cm, mean ± SD	168.1 ± 6.2	180.9 ± 6.4	169.0 ± 5.6	181.7 ± 6.0	167.5 ± 6.4	179.9 ± 6.8	168.6 ± 5.2	182.0 ± 7.0
Body mass index, mean ± SD	22.8 ± 2.6	24.1 ± 3.1	22.4 ± 2.5	23.5 ± 2.7	23.2 ± 3.0	24.2 ± 2.5	21.7 ± 2.4	23.5 ± 2.7
Gainful employment, %	95	94	93	96	98	99	100	95
Educated at university level, %	78	74	82	80	71	66	73	75
An income above 25.000 SEK** a month, %	60	73	58	72	36	69	61	80
Participant and both parents born in Sweden, %	79	86	82	84	80	89	69	87
Having a driver's licence, %	94	96	92	91	90	95	91	97
Usually access to a car, %	74	82	58	52	74	86	76	80
Leaving home 7-9 a.m. to cycle to work, %	72	67	84	85	68	61	81	82
Number of bicycle-commuting trips per year***, mean ± SD	240.7 ± 122.1	280.1 ± 135.7	317.7 ± 136.6	320.8 ± 117.3	265.8 ± 131.4	271.0 ± 141.4	301.4 ± 136.9	349.7 ± 84.1
Overall physical health either good or very good, %	86	86	80	76	82	80	97	93
Overall mental health either good or very good, %	83	86	84	80	80	84	91	88

Values are based on self-reports.

*Both I&S = those who bicycle-commuted in both the inner urban and suburban areas, *Only I *= those who bicycle-commuted in only the inner urban area and *Only S *= those who bicycle-commuted in only the suburban area.

**SEK = Swedish crown/krona, year 2005: €1 ≈ 9 SEK; US$1 ≈ 8 SEK.

***The number of bicycle-commuting trips per year is based on 239 women and 218 men in *Both I&*S, 161 women and 63 men in *Only I *and 316 women and 129 men in *Only S *and 18 women and 43 men in the street-recruited participant group. The low response rate is due to missing values in one or more of the 12 month leading to exclusion in the sum score.

### Street-recruited participants

We recruited participants while they were walking or bicycling into or in the inner urban area of Greater Stockholm, Sweden. The original purpose of the recruitment was to study the reliability of the PACS Q1 and Q2 questionnaires. This recruitment strategy was considered to represent the population of active commuters at that period of recruitment with greater certainty than the advertisement strategy. We therefore used these participants for representativity comparisons with advertisement-recruited participants.

The street-recruited participants were approached between 7 and 9 a.m. in mid-November 2005 as they either slowed down at one of four bridges or stopped at a traffic light on one arterial road. For geographical reasons, three of these places of recruitment (two bridges and one arterial road) were focal centres for active commuters entering the inner urban area of Stockholm from three different parts of the surrounding suburban areas. People living in these three different suburban areas represent slightly different sociodemographic characteristics. An invitation to participate together with a reply coupon was handed to 589 individuals. Inclusion criteria were the same as for advertisement-recruited participants. Overall, 214 coupons were returned in due time. The participants were then divided into two subgroups. One group (n = 114) was mainly used for a test-retest study of the PACS Q1 and one group (n = 100) was mainly used for a test-retest study of the PACS Q2. Test and retest questionnaires were sent to the participants during November and December 2005. Thereafter, in a crossover manner, the participants received the questionnaire that they did not respond to initially. The participants received a lottery ticket and a cycling map as a token of gratitude after returning the questionnaires. Cyclists and dual-mode performers who responded to the PACS Q2 at least once were selected for this study. We have used data from participants who bicycle-commuted in both inner urban and suburban areas. After cleansing and editing the data, 93 participants (women, n = 33, 35%) were included in the analyses. For further descriptive characteristics of the participants, see Table 1.

Some of the street-recruited participants took part in our previous study [9]. In this study, however, the number of participants has been increased from 44 to 93, which has been made possible by the crossover design described above.

### Experts

An expert panel of employees of the Municipality of Stockholm was assembled in September 2009 to assess the inner urban and suburban route environments of Greater Stockholm. Thirty-two relevant individuals were chosen to be part of the expert panel. They received a questionnaire with a modified version of the ACRES assessing bicyclists' perceptions. One item, *short or long*, was not included. The experts were asked to assess the overall route environments for cyclists commuting in Greater Stockholm and for the whole group of commuting cyclists, and the inner urban and suburban areas separately. Twenty-eight experts returned the questionnaire and data from 24 (women, n = 11) could be included in the analyses. Based on self-reports, their age was (mean ± SD) 43.8 ± 9.2 and the majority had a drivers licence (96%); usually had access to a car (83%); were educated at the university level (100%); and had an income of above 25000 SEK a month (100%; SEK = Swedish crown/krona, year 2009: €1 ≈ 10 SEK; US$1 ≈ 7 SEK). Ten usually commuted to work by bicycle all the year round and 4 during the spring to autumn months.

A more detailed description of the expert panel and the data used has been reported previously [9]. In this study we have only used the experts' mean values for the sizes of differences between perceptions of the inner urban and suburban route environments, rated using the modified version of the ACRES, for comparisons with the advertisement-recruited participants' corresponding values.

### Existing objective measurements

Differences between inner urban and suburban environments of Greater Stockholm, corresponding to the four ACRES items: *exhaust fumes*, *noise*, *congestion: all types of vehicles *and *greenery*, were used for comparisons of the direction of differences. Existing objective measurements showed higher levels for the inner urban environments than for the suburban environments corresponding to the items: *exhaust fumes *[12], *noise *[13] and *congestion: all types of vehicles *[14-16]. The opposite, higher levels for the suburban environments than for the inner urban environments, was shown corresponding to the item *greenery *[17]. A more detailed description of the existing objective measurements has been reported previously [9].

### The Physically Active Commuting in Greater Stockholm Questionnaire (PACS Q)

The PACS Q1 and PACS Q2 are self-administered questionnaires in Swedish, based on self-reports. They include about 35 and 70 items, respectively, comprising descriptive characteristics of participants and different aspects of active commuting. The PACS Q2 includes the ACRES.

#### Measure of descriptive characteristics

Data on sex, age, weight, height, employment, and number of bicycle-commuting trips per month were obtained from the PACS Q1. The body mass index (BMI) was calculated by dividing body weight by height squared (kg/m^2^). Active commuting trips per year were calculated by adding each of the 12 months' average trip frequency per week, dividing by 12 and multiplying by 52. Education levels, income, ethnicity, having a driver's licence, having access to a car, time leaving home to cycle to work, and overall physical and mental health were obtained from the PACS Q2.

#### The Active Commuting Route Environment Scale (ACRES)

The ACRES consists of 18 items for the assessment of bicyclists' perceptions of their self-chosen commuting route environment potentially associated with active commuting. A more detailed description of the development of the ACRES, its items, and its validity and reliability has been reported elsewhere [9]. The ACRES was characterized by considerable criterion-related validity and reasonable test-retest reproducibility, assessed on a street-recruited sample.

Each item considers the inner urban area of Stockholm, the capital of Sweden, and the suburban as well as rural areas surrounding it, within Stockholm County, separately. The questionnaire instructions include a drawn map that distinguishes the inner urban area from the surrounding areas. The participants are asked to differentiate between their experiences when their active commuting route is in the inner urban area and when it is in the surrounding suburban as well as rural areas. All items have two identical parallel response lines. One line refers to the inner urban area and the other to the suburban as well as rural areas. If the participants cycle or walk in both areas, they are asked to mark both lines. If the participants, for instance, first cycle in the southern suburban area, then cross into the inner urban area and finish their route in the northern suburban area, they are asked to give an average rating for both suburban areas of the route.

To simplify understanding, the items for the assessment of bicyclists' perceptions have been divided into: (a) the physical environment; (b) the traffic environment; and (c) the social environment. The following items are included in the physical environment: *bicycle paths*, *greenery*, *ugly or beautiful*, *course of the route*, *hilliness*, *red lights *and *short or long*. They represent non-moving aspects. The following items are included in the traffic environment: *exhaust fumes*, *noise*, *flow of motor vehicles*, *speeds of motor vehicles*, *speeds of bicyclists*, *congestion: all types of vehicles *and *congestion: bicyclists*. They represent moving aspects. The following item is included in the social environment: *conflicts*. It represents relationships between road users. All items are meant to operate independently. The remaining three items, namely, *on the whole*, *hinders or stimulates *and *traffic: unsafe or safe*, are regarded as outcome variables. All the other items are regarded as predictor variables believed to be potentially important for the outcome variables.

Fifteen-point response scales, with adjectival opposites, ranging from 1 to 15, corresponding to, for example, 'very low' and 'very high', are used, with the exception of one item. The item *bicycle paths *has an 11-point response scale ranging from 0% (0) to 100% (10). The fifteen-point response scales feature a numbered continuous line, i.e. whole numbers from 1 to 15, with number 8 as a neutral option in the middle, labelled, for example, 'neither low nor high'.

In the questionnaire instructions, the participants are asked to recall and rate their overall experience of their self-chosen route environments based on their active commuting to their place of work or study during the previous two weeks. At no point are the participants informed about the intent of the ACRES.

### Study area

#### Inner urban area

**Figure 1:**
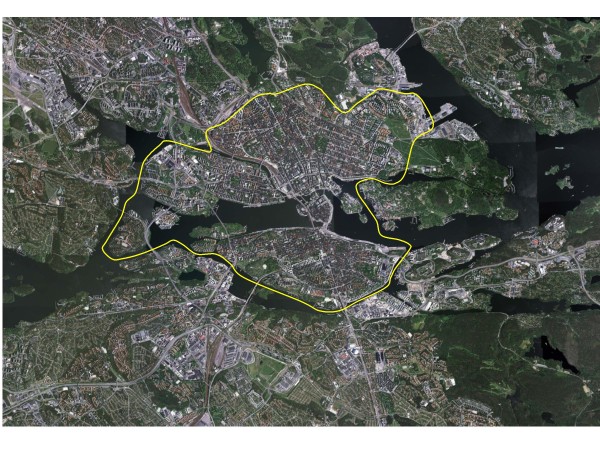
**Aerial view from 2005 over the more central parts of Greater Stockholm, Sweden**. The yellow line distinguishes the inner urban and the suburban as well as rural parts of the study area. For description of the characteristics of the study area, see Methods. (Copyright: Lantmäteriverket, Gävle, Sweden.)

The commuting route environments are located in the inner urban area of Stockholm, the capital of Sweden, situated in the centre of a metropolitan area with about 1.9 million inhabitants (Figure 1). This area constitutes the region's single core urban structure, with the centre situated where Lake Mälaren meets the Baltic Sea, thereby dividing the region into two main parts. The study area includes the city sections of 'Gamla stan' (the Old Town), Södermalm, Kungsholmen, Vasastan, Norrmalm and Östermalm (Figure 1). This is a predominantly built-up area, with blocks in a grid-like streetscape. The age of the buildings varies. The Old Town is from medieval times, whereas most parts of the built-up environment are predominantly a result of the architectural styles from the end of the 19^th ^and beginning of the 20^th ^century, with most buildings about five storeys high. The newest part of the city centre is north of the Old Town. The original buildings here were torn down during the 1950s and 1960s, and today the area includes modernistic architecture, including a few skyscrapers. In 2005 the residential density of the inner urban parts of the study area was approximately 13000 residents per square km [18].

The city has a number of waterfronts and islands, a number of both small and large parks, some alleys and esplanades. Most streets are void of trees or other forms of greenery. The natural landscape in the area is sediment-filled valleys as a part of the surrounding rift-valley landscape and raised archipelago landscape with eroded bedrocks after deglaciation. It is basically rather flat, but there are some dominant natural features as, for example, part of an esker, rising 40 metres above sea level in Vasastan, as well as a rather steep fault scarp in Södermalm. The road system also includes rather gentle slopes of infrequent moraine hills, normally not accounting for more than about 10 - 15 metres of elevation.

Two arterial highways pass through the inner urban area (Centralleden and Essingeleden), but they come into very little contact with cyclists or pedestrians. These are also the only roads, besides some tunnels, that do not permit cycling.

#### Suburban area

The commuting route environments are located in the suburban and rural areas of Greater Stockholm (Figure 1). These areas contain a mixture of residential areas, smaller industrial areas and managed forests as well as agricultural land. The residential areas either comprise predominantly single houses or constitute more dense areas with multi-storey houses. The single houses were mainly built during the 1930s and onwards in different architectural styles, whereas the majority of the densely built-up areas were fashioned in a modernistic style after the Second World War and during the 1970s. The residential density of the landscape normally varies with the proximity to subway or commuter train stations, which have small centres near the stations. As an indication of residential density of the suburban parts of our study area, we have chosen the southern and westerns suburbs of the Municipality of Stockholm, and in 2005 this amounted to approximately 3500 and 2900 residents per square km, respectively [18].

Houses are generally placed as separate entities in the landscape, not in blocks, and the streets are not normally laid out in a grid-like streetscape. Instead, the main roads often follow old road networks formed during the agricultural period of the landscape.

Trees or other forms of greenery exist in gardens and between the multi-storey houses, but not in alleys bordering the streets. The settlements, as well as the roads and traffic zones, lie in former agricultural landscapes in the sediment-filled valleys in this rift valley landscape. Between the valleys, the bedrocks often rise in faults, which are mostly covered with coniferous forest. The bedrocks often protrude from the thin soil cover (moraine). Forest-dominated areas stretch from the rural areas towards and into the centre of the region, between settlements and traffic zones, like ten green wedges. Lakes, islands and the Baltic Sea are other components.

The valleys in this area are basically rather flat, but the road system also includes rather gentle slopes of infrequent moraine hills from the deglaciation, normally not accounting for more than about 10 - 15 metres of elevation. Some arterial highways pass through the landscape and do so with varying contact with cyclists and pedestrians.

### Statistical analyses

Questionnaire data were entered in the Statistical Package for the Social Sciences, version 17.0 (IBM SPSS Inc., Somer, NY). After that, we checked all entered data from the PACS Q2 for accuracy. Some participants were excluded, mainly because of incorrect or incomplete ACRES data. Participants with more than three missing ACRES values for cyclists were excluded.

Percentages and mean scores ± 1 standard deviation (SD) were used to report the characteristics of the participants. The values of the ACRES items are presented as mean scores ± 1 SD or the standard error of difference (SEd). Analyses of differences between ratings of inner urban and suburban route environments (*Both I&S*, *Only I *and *Only S*) and between groups (*Both I&S *and *Only I *or *Only S*) were performed using paired or independent t-tests. Since the street-recruited participants provided a relatively small sample size, we initially also used a non-parametric test (Wilcoxon's signed-ranks test) to analyse the differences between inner urban and suburban route environments. The results did not differ and we have therefore chosen to present only the results from the parametric tests. Linear regression analysis and Pearson's correlation coefficient were used to assess the agreement between mean scores (between different mean values for the experts and the advertisement-recruited participants [*Both I&S*], as well as between the street- and the advertisement-recruited participants [*Both I&S*]). The estimated linear regression lines were not considered to deviate significantly if the 95% confidence intervals included 1.0 for the slope and 0.0 for the y-intercept (line of identity: slope = 1.0 and y-intercept = 0.0). We have chosen to place the experts' and the street-recruited participants' values on the x-axis. All linear regression analyses were, however, initially performed with the advertisement-recruited participants' values placed alternately on the y- and x-axes. The interpretation of similarity to the line of identity only differed for two cases. However, these differences were of a very minor magnitude. A statistical level corresponding to at least p ≤ 0.05 has been used to indicate significance. All the values from the ACRES items rated by the advertisement-recruited participants (*Both I&S*, *Only I *and *Only S*) are used twice in the analyses. We have, therefore, chosen in those cases the lower statistical level of p ≤ 0.025, instead of p ≤ 0.05, to define significance, as a way of handling the problem of multiple comparisons using the same data.

We have performed separate analyses of men and women in all groups (*Both I&S*, *Only I*, *Only S *and street-recruited participants), except for the experts. The small sample size of experts did not allow separate analyses. The experts were, however, practically evenly distributed between men and women (women, n = 11, 46%). We therefore used the combined experts for the comparisons with the advertisement-recruited participants (*Both I&S*) separated into men and women. Differences between men and women were not analysed for significance due to the problem with multiple comparisons. For comparison between the advertisement-recruited (*Both I&S*) and the street-recruited participants, the men's and women's scores were combined to give a 'sex-neutral' mean for each rating of the ACRES items regarding absolute levels, distributions of values (means for the standard deviations), and sizes of differences between the inner urban and suburban route environments.

## Results

### Criterion-related validity: differences in ratings of inner urban and suburban route environments

Mean scores on all items of inner urban and suburban route environments for the advertisement-recruited participants who bicycle-commuted in both inner urban and suburban areas (*Both I&S*) are shown in Table 2. The ratings by the advertisement-recruited men and women, respectively, showed significantly higher values for the inner urban route environments than for the suburban route environments in the items *exhaust fumes*, *noise *and *congestion: all types of vehicles*. The opposite was observed for the item *greenery *(see Table 2). These findings corresponded with the directions of differences noted in the existing objective measurements (see Methods).

**Table 2:** Environment ratings by advertisement-recruited participants bicycle-commuting in both inner urban and suburban areas

	Women (n = 286-302)						Men (n = 247-253)					
		
	Inner urban		Suburban		Difference		Inner urban		Suburban		Difference	
						
Item	Mean ± SD	Min - max	Mean ± SD	Min - max	Mean ± SD	p-value	Mean ± SD	Min - max	Mean ± SD	Min - max	Mean ± SD	p-value
1. On the whole	8.49 ± 3.41	1 - 15	11.25 ± 2.82	2 - 15	-2.77 ± 3.51	0.000	8.49 ± 3.29	1 - 15	11.15 ± 2.68	3 - 15	-2.66 ± 3.56	0.000
2. Hinders or stimulates	9.13 ± 3.49	1 - 15	11.28 ± 2.93	1 - 15	-2.15 ± 3.76	0.000	8.92 ± 3.08	1 - 15	10.82 ± 2.86	1 - 15	-1.90 ± 3.36	0.000
3. Exhaust fumes	10.44 ± 3.24	1 - 15	7.53 ± 3.46	1 - 15	2.91 ± 4.12	0.000	9.02 ± 3.21	1 - 15	6.36 ± 3.28	1 - 15	2.66 ± 3.63	0.000
4. Noise	10.23 ± 3.11	1 - 15	7.67 ± 3.60	1 - 15	2.56 ± 3.96	0.000	8.74 ± 3.02	1 - 15	6.84 ± 3.29	1 - 15	1.90 ± 3.74	0.000
5. Flow of motor vehicles	11.43 ± 3.40	1 - 15	8.18 ± 3.94	1 - 15	3.25 ± 4.59	0.000	10.55 ± 3.67	1 - 15	7.66 ± 3.79	1 - 15	2.89 ± 4.70	0.000
6. Speeds of motor vehicles	9.74 ± 2.88	1 - 15	8.99 ± 3.32	1 - 15	0.74 ± 3.56	0.000	8.73 ± 2.77	1 - 15	8.79 ± 2.89	1 - 15	-0.06 ± 2.81	0.754
7. Speeds of bicyclists	9.94 ± 2.73	1 - 15	9.92 ± 2.59	1 - 15	0.03 ± 2.45	0.851	7.76 ± 2.84	1 - 15	8.06 ± 2.62	1 - 15	-0.29 ± 2.13	0.031
8. Congestion: all types of vehicles	10.72 ± 3.52	1 - 15	6.74 ± 3.42	1 - 15	3.98 ± 4.20	0.000	10.14 ± 3.42	1 - 15	5.46 ± 3.08	1 - 15	4.68 ± 4.08	0.000
9. Congestion: bicyclists	9.31 ± 3.81	1 - 15	6.26 ± 3.76	1 - 15	3.04 ± 4.35	0.000	8.64 ± 3.90	1 - 15	4.99 ± 3.29	1 - 15	3.65 ± 4.31	0.000
10. Conflicts	8.14 ± 3.82	1 - 15	5.44 ± 3.63	1 - 15	2.70 ± 4.00	0.000	8.56 ± 3.77	1 - 15	5.19 ± 3.47	1 - 15	3.37 ± 3.88	0.000
11. Bicycle paths*	6.37 ± 2.95	0 - 10	7.67 ± 2.35	0 - 10	-1.30 ± 3.75	0.000	5.90 ± 2.77	0 - 10	7.42 ± 2.30	0 - 10	-1.52 ± 3.41	0.000
12. Traffic: unsafe or safe	8.24 ± 3.89	1 - 15	11.29 ± 2.99	2 - 15	-3.05 ± 3.96	0.000	8.68 ± 3.55	1 - 15	11.55 ± 2.82	1 - 15	-2.87 ± 3.40	0.000
13. Greenery	7.09 ± 4.11	1 - 15	10.96 ± 3.43	1 - 15	-3.86 ± 4.68	0.000	6.72 ± 3.88	1 - 15	10.84 ± 2.98	1 - 15	-4.12 ± 4.29	0.000
14. Ugly or beautiful	9.88 ± 3.35	1 - 15	10.74 ± 3.08	1 - 15	-0.86 ± 4.12	0.000	9.84 ± 3.22	1 - 15	10.21 ± 2.99	1 - 15	-0.37 ± 4.01	0.145
15. Course of the route	6.98 ± 3.86	1 - 15	5.41 ± 3.51	1 - 15	1.57 ± 3.98	0.000	7.48 ± 4.01	1 - 15	5.60 ± 3.65	1 - 15	1.89 ± 3.60	0.000
16. Hilliness	5.20 ± 3.70	1 - 15	6.61 ± 4.16	1 - 15	-1.41 ± 3.79	0.000	5.07 ± 3.44	1 - 13	6.24 ± 3.83	1 - 15	-1.17 ± 2.91	0.000
17. Red lights	8.06 ± 4.62	1 - 15	3.98 ± 3.48	1 - 15	4.08 ± 5.20	0.000	8.25 ± 4.33	1 - 15	4.50 ± 3.46	1 - 15	3.75 ± 4.88	0.000
18. Short or long	6.78 ± 2.98	1 - 15	7.78 ± 3.02	1 - 15	-1.00 ± 3.95	0.000	6.95 ± 2.32	1 - 14	7.85 ± 2.71	1 - 14	-0.90 ± 3.10	0.000

*Minimal value = 0 and maximal value = 10.

**Figure 2:**
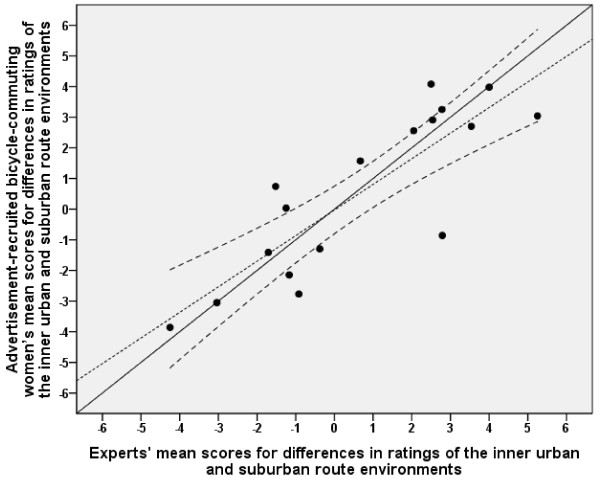
**Relationship between advertisement-recruited women and experts in differences between inner urban and suburban route environments**. The solid diagonal line represents the line of identity (slope = 1.0 and y-intercept = 0.0). The dotted line represents the linear regression line. The upper and lower dashed lines on either side of the linear regression line represent the 95% confidence interval (CI). The regression line (y = -0.03 [-0.80 - 0.75] + 0.84 [0.55 - 1.12] ×, [95% CI]) did not deviate significantly from the line of identity. Note that the means are sometimes minus values. The interpretation of the y-intercept could therefore be misleading. The 95% CI for the slope therefore gives a better picture, showing that the linear regression line did not deviate significantly from the line of identity. Pearson's correlation was 0.85.

**Figure 3:**
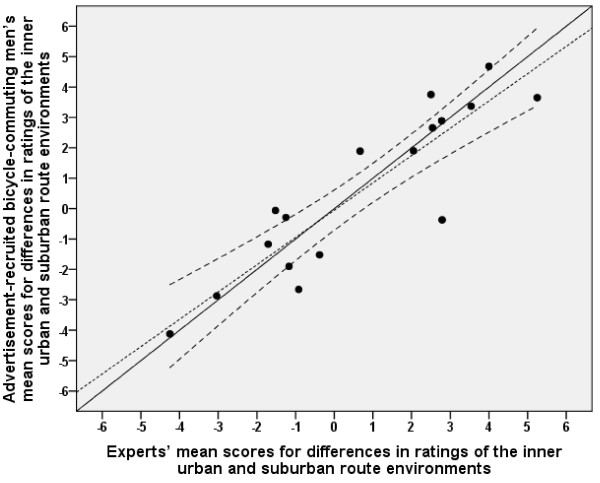
**Relationship between advertisement-recruited men and experts in differences between inner urban and suburban route environments**. The solid diagonal line represents the line of identity (slope = 1.0 and y-intercept = 0.0). The dotted line represents the linear regression line. The upper and lower dashed lines on either side of the linear regression line represent the 95% confidence interval (CI). The regression line (y = -0.05 [-0.71 - 0.61] + 0.90 [0.66 - 1.14] ×, [95% CI]) did not deviate significantly from the line of identity. Note that the means are sometimes minus values. The interpretation of the y-intercept could therefore be misleading. The 95% CI for the slope therefore gives a better picture, showing that the linear regression line did not deviate significantly from the line of identity. Pearson's correlation was 0.90.

The sizes of differences in ratings of inner urban and suburban route environments between the experts and the advertisement-recruited men and women, respectively, matched reasonably well (see Figures 2 and 3, and Table 2 for the values of the advertisement-recruited participants). The regression lines did not deviate significantly from the line of identity. The values correlated well (women, *r *= 0.85, and men, *r *= 0.90).

### Representativity: relationships between ratings of advertisement-recruited and street-recruited participants

**Figure 4:**
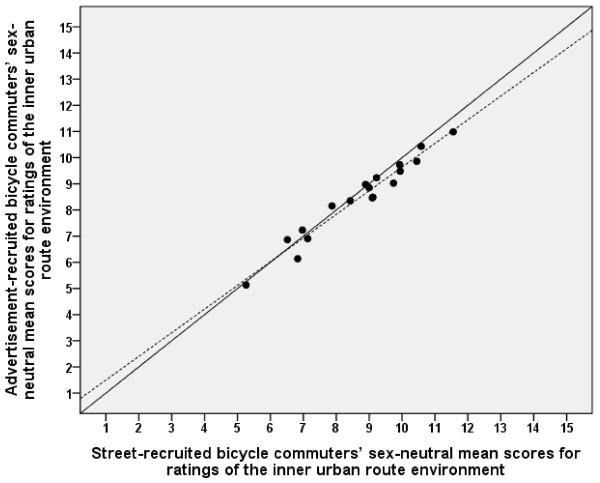
**Relationship between advertisement-recruited and street-recruited participants' ratings of inner urban route environments**. The mean values are expressed as sex-neutral means. The solid diagonal line represents the line of identity (slope = 1.0 and y-intercept = 0.0). The dotted line represents the linear regression line. The regression line (y = 0.59 [-0.30 - 1.49] + 0.90 [0.80 - 1.01] ×, [95% confidence interval]) did not deviate significantly from the line of identity. Pearson's correlation was 0.98.

**Figure 5:**
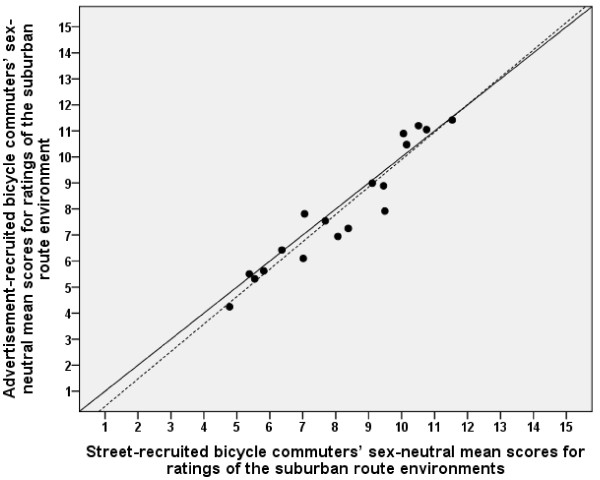
**Relationship between advertisement-recruited and street-recruited participants' ratings of suburban route environments**. The mean values are expressed as sex-neutral means. The solid diagonal line represents the line of identity (slope = 1.0 and y-intercept = 0.0). The dotted line represents the linear regression line. The regression line (y = -0.63 [-2.08 - 0.81] + 1.05 [0.88 - 1.22] ×, [95% confidence interval]) did not deviate significantly from the line of identity. Pearson's correlation was 0.96.

Mean scores on all items of inner urban and suburban route environments for the street-recruited participants are shown in Table 3. The sex-neutral means corresponded well between the advertisement-recruited (*Both I&S*) and the street-recruited participants for both the inner urban and the suburban route environments (see Figures 4 and 5). The regression lines did not deviate significantly from the line of identity. The values correlated well (inner urban, *r *= 0.98, and suburban, *r *= 0.96).

**Table 3:** Environment ratings by street-recruited participants bicycle-commuting in both inner urban and suburban areas

	Women (n = 32-33)						Men (n = 58-60)					
		
	Inner urban		Suburban		Difference		Inner urban		Suburban		Difference	
						
Item	Mean ± SD	Min - max	Mean ± SD	Min - max	Mean ± SD	p-value	Mean ± SD	Min - max	Mean ± SD	Min - max	Mean ± SD	p-value
1. On the whole	10.03 ± 2.55	4 - 14	10.78 ± 2.95	5 - 15	-0.75 ± 2.87	0.150	8.19 ± 3.61	1 - 15	10.24 ± 2.77	4 - 15	-2.05 ± 3.59	0.000
2. Hinders or stimulates	10.58 ± 2.80	3 - 14	10.85 ± 3.03	1 - 15	-0.27 ± 3.10	0.616	8.90 ± 3.29	3 - 15	10.66 ± 2.65	4 - 15	-1.76 ± 3.47	0.000
3. Exhaust fumes	10.18 ± 3.14	2 - 15	8.61 ± 2.84	3 - 14	1.58 ± 3.84	0.025	9.68 ± 3.00	3 - 15	7.52 ± 3.39	1 - 15	2.17 ± 3.66	0.000
4. Noise	10.06 ± 3.17	3 - 15	8.24 ± 3.10	1 - 14	1.82 ± 3.96	0.013	9.83 ± 2.66	4 - 14	8.52 ± 3.46	1 - 14	1.32 ± 3.59	0.006
5. Flow of motor vehicles	11.73 ± 3.46	3 - 15	9.45 ± 3.75	1 - 15	2.27 ± 4.03	0.003	11.38 ± 2.63	4 - 15	9.53 ± 3.95	1 - 15	1.84 ± 3.75	0.000
6. Speeds of motor vehicles	9.53 ± 2.59	3 - 15	9.59 ± 2.34	3 - 14	-0.06 ± 1.50	0.815	8.92 ± 2.77	1 - 15	9.31 ± 2.76	1 - 15	-0.39 ± 2.28	0.195
7. Speeds of bicyclists	10.15 ± 2.74	4 - 15	9.67 ± 2.57	5 - 15	0.48 ± 2.54	0.281	7.85 ± 2.04	4 - 14	8.55 ± 2.00	1 - 14	-0.70 ± 1.84	0.005
8. Congestion: all types of vehicles	10.63 ± 3.14	2 - 15	7.50 ± 2.63	2 - 12	3.13 ± 3.38	0.000	10.53 ± 3.10	2 - 15	6.53 ± 3.11	1 - 15	4.00 ± 3.69	0.000
9. Congestion: bicyclists	9.61 ± 4.03	2 - 15	6.48 ± 3.35	1 - 13	3.12 ± 4.58	0.000	8.17 ± 3.81	1 - 15	5.15 ± 3.27	1 - 12	3.02 ± 3.93	0.000
10. Conflicts	8.42 ± 3.89	1 - 14	5.70 ± 3.65	1 - 13	2.73 ± 3.78	0.000	8.43 ± 3.97	1 - 15	5.38 ± 3.37	1 - 13	3.05 ± 3.98	0.000
11. Bicycle paths*	7.22 ± 2.27	1 - 10	7.91 ± 2.18	2 - 10	-0.69 ± 3.25	0.240	6.44 ± 2.47	0 - 10	7.46 ± 2.49	1 - 10	-1.02 ± 3.50	0.029
12. Traffic: unsafe or safe	9.42 ± 3.47	3 - 15	11.45 ± 2.59	8 - 15	-2.03 ± 3.04	0.001	8.77 ± 3.22	1 - 15	11.62 ± 2.58	5 - 15	-2.85 ± 3.44	0.000
13. Greenery	8.12 ± 3.45	2 - 15	9.91 ± 3.47	1 - 15	-1.79 ± 4.49	0.029	6.14 ± 3.42	1 - 15	10.20 ± 3.18	1 - 15	-4.07 ± 4.48	0.000
14. Ugly or beautiful	11.42 ± 2.96	5 - 15	11.12 ± 3.04	4 - 15	0.30 ± 3.79	0.649	9.47 ± 3.32	3 - 15	9.18 ± 3.29	3 - 15	0.28 ± 4.78	0.648
15. Course of the route	6.59 ± 3.79	1 - 13	5.38 ± 3.66	1 - 13	1.22 ± 2.62	0.013	7.35 ± 3.24	2 - 14	5.38 ± 3.38	1 - 14	1.97 ± 3.96	0.000
16. Hilliness	5.82 ± 3.81	1 - 13	6.21 ± 3.87	1 - 14	-0.39 ± 3.40	0.510	4.70 ± 3.30	1 - 12	6.52 ± 3.88	1 - 14	-1.82 ± 4.18	0.001
17. Red lights	8.09 ± 4.38	1 - 15	5.33 ± 3.59	1 - 15	2.76 ± 3.64	0.000	7.65 ± 3.93	1 - 14	4.23 ± 3.38	1 - 13	3.42 ± 4.61	0.000
18. Short or long	6.58 ± 2.37	1 - 10	6.91 ± 3.22	1 - 12	-0.33 ± 4.07	0.641	6.45 ± 2.22	2 - 10	7.20 ± 2.48	1 - 12	-0.75 ± 3.14	0.069

*Minimal value = 0 and maximal value = 10.

In general, the sex-neutral means for the standard deviations corresponded reasonably well between the advertisement- and the street-recruited participants for both the inner urban and the suburban route environment. The regression line for the inner urban route environment deviated slightly from the line of identity (y = 0.74 [0.17 - 1.30] + 0.85 [0.67 - 1.03] ×, [figures within brackets represent the 95% confidence interval]). The regression line for the suburban route environment did not deviate significantly from the line of identity (y = 0.60 [-0.01 - 1.21] + 0.84 [0.65 - 1.04] ×). The values correlated well (inner urban, *r *= 0.93, and suburban, *r *= 0.92).

In the street-recruited participants, significant differences were seen between ratings of inner urban and suburban route environments in 10 of 18 items rated by the women and in 15 of 18 items rated by the men. Correspondence in both significance and direction of the differences was seen between the advertisement- and the street-recruited participants in 10 of the 18 items for the women and in 14 of the 18 items for the men (see Tables 2 and 3).

**Figure 6:**
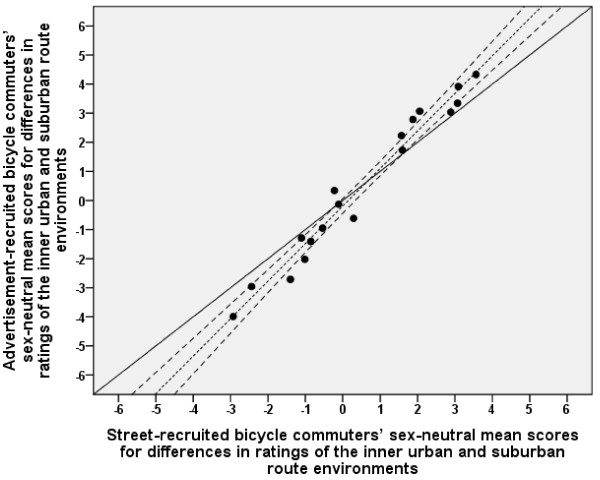
**Relationship between advertisement-recruited and street-recruited participants in differences between inner urban and suburban route environments**. The mean values are expressed as sex-neutral means. The solid diagonal line represents the line of identity (slope = 1.0 and y-intercept = 0.0). The dotted line represents the linear regression line. The upper and lower dashed lines on either side of the linear regression line represent the 95% confidence interval (CI). The regression line (y = -0.19 [-0.44 - 0.06] + 1.29 [1.17 - 1.42] ×, [95% CI]) deviated slightly from the line of identity. Note that the means are sometimes minus values. The interpretation of the y-intercept could therefore be misleading. The 95% CI for the slope therefore gives a better picture, showing that the linear regression line deviated slightly from the line of identity. Pearson's correlation was 0.98.

The sizes of differences between ratings of the inner urban and suburban route environments for the advertisement- and the street-recruited participants are shown in Tables 2 and 3, respectively. The sex-neutral means corresponded reasonably well between the advertisement- and the street-recruited participants (see Figure 6). The regression line deviated slightly from the line of identity. The values correlated well (*r *= 0.98).

### Commuting route environment profiles: comparisons between the inner urban and suburban areas as well as between the subgroups

**Figure 7:**
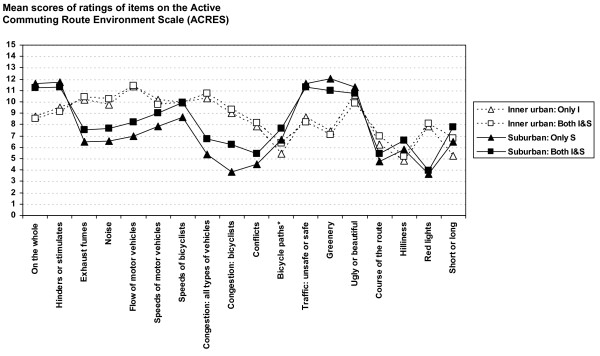
**Commuting route environment profiles for women cycling in the inner urban and suburban areas**. Advertisement-recruited participants: *Both I&S *= those who bicycle-commuted in both the inner urban and suburban areas, *Only I *= those who bicycle-commuted in only the inner urban area and *Only S *= those who bicycle-commuted in only the suburban area. Unfilled symbols represent ratings of the inner urban route environments. Filled symbols represent the suburban route environments. *Minimal value = 0 and maximal value = 10.

**Figure 8:**
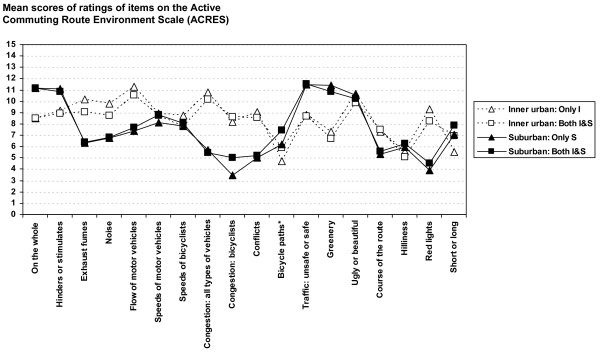
**Commuting route environment profiles for men cycling in the inner urban and suburban areas**. Advertisement-recruited participants: *Both I&S *= those who bicycle-commuted in both the inner urban and suburban areas, *Only I *= those who bicycle-commuted in only the inner urban area and *Only S *= those who bicycle-commuted in only the suburban area. Unfilled symbols represent ratings of the inner urban route environments. Filled symbols represent the suburban route environments. *Minimal value = 0 and maximal value = 10.

Commuting route environment profiles, based on mean scores on all items regarding women's and men's ratings of inner urban and suburban route environments for the advertisement-recruited participants (*Both I&S*, i.e. those who bicycle-commuted in both inner urban and suburban areas, *Only I*, i.e. those who bicycle-commuted in only an inner urban area and *Only *S, i.e. those who bicycle-commuted in only a suburban area) are shown in Figure 7 for women and in Figure 8 for men.

Mean scores on all items of inner urban and suburban route environments for *Both I&S *are shown in Table 2. Significant differences were seen between ratings of inner urban and suburban route environments on 17 of 18 items rated by the women and on 15 of 18 items rated by the men. Correspondence between men and women in both the significance and direction of the differences was noted in 15 of the 18 items.

Mean scores on all items of inner urban and suburban route environments for *Only I *and *Only S *are shown in Table 4. Significant differences were seen between ratings of inner urban and suburban route environments on all of 18 items rated by *Only I *women and *Only S *women, and on 15 of 18 items rated by *Only I *men and *Only S *men. Correspondence between men and women in both the significance and direction of the differences was noted in 15 of the 18 items.

**Table 4:** Environment ratings by advertisement-recruited participants bicycle-commuting in the inner urban or suburban area

	Women				Men			
		
	Only I* (n = 194-197)	Only S* (n = 394-399)	Difference		*Only I *(n = 74-75)	*Only S *(n = 151-153)	Difference	
						
Item	Mean ± SD	Mean ± SD	Mean ± SEd**	p-value	Mean ± SD	Mean ± SD	Mean ± SEd	p-value
1. On the whole	8.72 ± 3.35	11.63 ± 2.75	-2.92 ± 0.28	0.000	8.47 ± 3.33	11.14 ± 2.66	-2.67 ± 0.44	0.000
2. Hinders or stimulates	9.52 ± 3.52	11.73 ± 2.70	-2.21 ± 0.29	0.000	9.15 ± 2.78	11.08 ± 2.83	-1.94 ± 0.40	0.000
3. Exhaust fumes	10.16 ± 2.86	6.47 ± 3.75	3.69 ± 0.28	0.000	10.15 ± 2.74	6.35 ± 3.40	3.79 ± 0.42	0.000
4. Noise	9.78 ± 2.78	6.56 ± 3.75	3.21 ± 0.27	0.000	9.79 ± 2.78	6.78 ± 3.19	3.00 ± 0.43	0.000
5. Flow of motor vehicles	11.35 ± 2.93	6.99 ± 4.13	4.36 ± 0.29	0.000	11.27 ± 2.69	7.35 ± 3.55	3.92 ± 0.42	0.000
6. Speeds of motor vehicles	10.16 ± 2.63	7.81 ± 3.34	2.35 ± 0.25	0.000	8.91 ± 2.73	8.14 ± 3.15	0.76 ± 0.43	0.075
7. Speeds of bicyclists	9.91 ± 2.42	8.64 ± 2.51	1.27 ± 0.22	0.000	8.77 ± 2.59	7.80 ± 1.94	0.97 ± 0.34	0.005
8. Congestion: all types of vehicles	10.33 ± 2.97	5.35 ± 3.55	4.98 ± 0.28	0.000	10.77 ± 2.70	5.69 ± 3.23	5.09 ± 0.41	0.000
9. Congestion: bicyclists	9.01 ± 3.33	3.84 ± 2.98	5.17 ± 0.27	0.000	8.20 ± 3.67	3.50 ± 2.52	4.70 ± 0.47	0.000
10. Conflicts	7.81 ± 3.60	4.49 ± 3.44	3.32 ± 0.30	0.000	9.08 ± 3.38	5.01 ± 3.54	4.07 ± 0.49	0.000
11. Bicycle paths***	5.45 ± 2.76	6.65 ± 2.84	-1.20 ± 0.24	0.000	4.73 ± 2.88	6.18 ± 2.78	-1.45 ± 0.40	0.000
12. Traffic: unsafe or safe	8.66 ± 3.59	11.61 ± 3.10	-2.94 ± 0.30	0.000	8.81 ± 3.54	11.46 ± 2.76	-2.64 ± 0.47	0.000
13. Greenery	7.43 ± 4.17	12.04 ± 2.88	-4.61 ± 0.33	0.000	7.33 ± 3.71	11.39 ± 2.84	-4.06 ± 0.49	0.000
14. Ugly or beautiful	10.62 ± 3.32	11.28 ± 2.73	-0.66 ± 0.27	0.017	10.68 ± 2.93	10.53 ± 2.68	0.15 ± 0.39	0.700
15. Course of the route	6.25 ± 3.43	4.74 ± 3.45	1.51 ± 0.30	0.000	7.29 ± 3.86	5.31 ± 3.18	1.99 ± 0.48	0.000
16. Hilliness	4.81 ± 3.46	5.78 ± 3.91	-0.97 ± 0.32	0.002	5.56 ± 3.48	5.93 ± 3.84	-0.37 ± 0.53	0.477
17. Red lights	7.87 ± 3.98	3.62 ± 3.52	4.26 ± 0.33	0.000	9.27 ± 3.61	3.90 ± 3.20	5.36 ± 0.47	0.000
18. Short or long	5.26 ± 2.64	6.46 ± 3.37	-1.20 ± 0.25	0.000	5.52 ± 2.75	7.08 ± 2.87	-1.56 ± 0.40	0.000

*Only I = those who bicycle-commuted in only the inner urban area, and *Only S *= those who bicycle-commuted in only the suburban area.

**SEd = standard error of difference.

***Minimal value = 0 and maximal value = 10.

Altogether (*Both I&S*, *Only I *and *Only S*, categorized as men and women), both the significance and direction of the differences between the inner urban and the suburban route environments corresponded in 14 of the 18 items. For all of these four groups, significantly higher values for the inner urban than for the suburban route environments were seen for the items: *exhaust fumes*, *noise*, *flow of motor vehicles*, *congestion: all types of vehicles*, *congestion: bicyclists*, *conflicts*, *course of the route *and *red lights*. The opposite, significantly higher values for the suburban route environments than for the inner urban ones, was seen for the items: *on the whole*, *hinders or stimulates*, *bicycle paths*, *traffic: unsafe or safe*, *greenery *and *short or long *(see Tables 2 and 4).

A high degree of resemblance was noted in the ratings of route environments in each area regardless of whether the participants bicycled in only one area (inner urban or suburban) or in both of these areas. Although of small magnitude, statistically significant differences in ratings of the inner urban route environments between *Both I&S *and *Only I *were seen in 3 of 18 items for the women and in 5 of 18 items for the men. Significant differences in ratings of the suburban route environments between *Both I&S *and *Only S *were seen in 14 of 18 items for women and 3 of 18 items for men (see Table 5).

**Table 5:** Differences in ratings of route environments by subgroups of advertisement-recruited participants

	Inner urban: Difference: Only I* and Both I&S*				Suburban: Difference: Only S* and *Both I&S*			
		
	Women		Men		Women		Men	
				
Item	Mean ± SEd**	p-value	Mean ± SEd	p-value	Mean ± SEd	p-value	Mean ± SEd	p-value
1. On the whole	0.24 ± 0.32	0.439	-0.02 ± 0.44	0.965	0.38 ± 0.22	0.081	-0.01 ± 0.28	0.981
2. Hinders or stimulates	0.39 ± 0.32	0.230	0.24 ± 0.40	0.554	0.45 ± 0.22	0.038	0.27 ± 0.29	0.359
3. Exhaust fumes	-0.28 ± 0.28	0.318	1.12 ± 0.41	0.006	-1.06 ± 0.27	0.000	-0.01 ± 0.34	0.981
4. Noise	-0.45 ± 0.27	0.099	1.05 ± 0.39	0.008	-1.09 ± 0.28	0.000	-0.05 ± 0.33	0.872
5. Flow of motor vehicles	-0.09 ± 0.29	0.749	0.70 ± 0.39	0.073	-1.19 ± 0.31	0.000	-0.32 ± 0.38	0.405
6. Speeds of motor vehicles	0.42 ± 0.25	0.098	0.18 ± 0.36	0.629	-1.18 ± 0.25	0.000	-0.65 ± 0.31	0.036
7. Speeds of bicyclists	-0.03 ± 0.24	0.899	1.01 ± 0.37	0.006	-1.28 ± 0.20	0.000	-0.25 ± 0.23	0.270
8. Congestion: all types of vehicles	-0.40 ± 0.29	0.173	0.66 ± 0.38	0.082	-1.38 ± 0.27	0.000	0.23 ± 0.32	0.479
9. Congestion: bicyclists	-0.30 ± 0.32	0.358	-0.44 ± 0.51	0.381	-2.42 ± 0.26	0.000	-1.49 ± 0.29	0.000
10. Conflicts	-0.33 ± 0.34	0.337	0.52 ± 0.48	0.287	-0.95 ± 0.27	0.001	-0.16 ± 0.36	0.654
11. Bicycle paths***	-0.93 ± 0.26	0.000	-1.15 ± 0.37	0.002	-1.01 ± 0.20	0.000	-1.24 ± 0.27	0.000
12. Traffic: unsafe or safe	0.43 ± 0.35	0.218	0.13 ± 0.47	0.781	0.32 ± 0.23	0.173	-0.09 ± 0.29	0.749
13. Greenery	0.33 ± 0.38	0.378	0.61 ± 0.51	0.226	1.08 ± 0.24	0.000	0.56 ± 0.30	0.065
14. Ugly or beautiful	0.73 ± 0.31	0.017	0.84 ± 0.42	0.044	0.53 ± 0.22	0.018	0.32 ± 0.29	0.280
15. Course of the route	-0.72 ± 0.34	0.033	-0.19 ± 0.52	0.715	-0.68 ± 0.27	0.011	-0.29 ± 0.35	0.405
16. Hilliness	-0.39 ± 0.33	0.236	0.49 ± 0.45	0.282	-0.83 ± 0.31	0.007	-0.31 ± 0.39	0.435
17. Red lights	-0.19 ± 0.39	0.632	1.02 ± 0.50	0.043	-0.36 ± 0.27	0.176	-0.60 ± 0.34	0.085
18. Short or long	-1.52 ± 0.26	0.000	-1.43 ± 0.35	0.000	-1.32 ± 0.24	0.000	-0.77 ± 0.29	0.008

*Both I&S = those who bicycle-commuted in both the inner urban and suburban areas, *Only I *= those who bicycle-commuted in only the inner urban area and *Only S *= those who bicycle-commuted in only the suburban area.

**SEd = standard error of difference.

***Minimal value = 0 and maximal value = 10.

## Discussion

This study was conducted to expand the knowledge of the ACRES regarding methodological issues as well as to compare the commuting route environment profiles of inner urban and suburban areas and to interpret the consequences in terms of bikeability, as defined below.

The main results show: (1) a considerable criterion-related validity of the ACRES; (2) general concordance between advertisement- and street-recruited bicycle commuters regarding both mean ratings of different items, distribution of the values and in the sizes and directions of differences in ratings between route environments; and (3) clear differences in commuting route environment profiles between the inner urban and suburban areas, demonstrating a higher level of bikeability of route environments in the suburban areas. The evidence for these interpretations of the results will be discussed below.

### Criterion-related validity

The first aim of this study dealt with a criterion-related validity assessment of the ACRES. In evaluating this issue, we have combined findings from all three Results sections. The first way of illuminating this issue was based on whether or not differences between the inner urban and the suburban areas were reflected by differences in perceptions of the two environments. And this was the case. First, directions of the ratings of the four ACRES items, *exhaust fumes*, *noise*, *congestion: all types of vehicles *and *greenery*, by both advertisement-recruited (*Both I&S*) and street-recruited men and women bicycle-commuting in inner urban and suburban areas, corresponded with the directions of the existing objective measurements. Interestingly, the same findings were noted when the ratings for the groups of advertisement-recruited men and women who only cycled in either the inner urban (*Only I*) or the suburban (*Only S*) area were compared. Second, the sizes of the differences in ratings of the route environments by *Both I&S*, men and women, corresponded reasonably well with the ratings of the experts. These results are in agreement with our previous findings [9], thus further supporting and strengthening the criterion-related validity of the ACRES. Our previous study was based on a smaller sample of street-recruited participants. In this study, we used both a larger sample of participants recruited through morning newspapers, and an enlarged sample of street-recruited participants. The similar results therefore also strengthen the external validity.

A third dimension of criterion-related validity relates to the question of whether ratings of an area would be affected by whether the participants had also experienced and rated another area. It was possible to evaluate this by comparing the ratings of commuters in *Only I *or *Only S *and *Both I&S*. Since the inner urban route environment setting is rather discrete and homogeneous in nature, compared to the suburban setting, which has a gradient all the way from close to the inner urban qualities in some suburbs near the inner urban area to rural qualities, we thought that the best comparison in this respect would be to compare the ratings of the inner urban area between *Both I&S *and *Only I*. The deviances noted between the two female groups are remarkably small (mean absolute difference: 0.45 ± 0.35, n = 18). The same is true of the male groups (mean absolute difference: 0.66 ± 0.41, n = 18; cf. Table 4 and Figures 7 and 8). Significant differences exist in some cases, but the magnitudes are small. We therefore interpret this result as a sign of robustness of the ACRES. In our mind, these results further strengthen the criterion-related validity of the ACRES. Thus, summing up these, we regard the criterion-related validity of the ACRES to be considerable.

Interestingly, however, significant differences in most of the items were seen in ratings of the suburban route environments between women *Both I&S *and *Only S*. Note that the differences that generate statistical significance are small (mean absolute difference: 1.14 ± 0.44, n = 14), considering that the response scales have 15 points, and that this reflects that the sample size was fairly large. Still, this finding is worth considering, particularly since the corresponding deviance is not noted in the males. At present, we can only speculate about possible explanations. In our minds, a plausible explanation is that *Only S *women's suburban areas are located more distant from the inner urban areas and therefore might constitute a slightly different suburban area as compared to *Both I&S *women's suburban settings. Differences in the spatial distribution of workplaces for men and women [19], as well as differences in the distances covered by them in their bicycle-commuting [8], support such an interpretation. The findings indicate that there is much to be learned about the actual route taken, in relation to route environments, and potential differences between men and women in this respect.

Options for future studies on the validity and other psychometric properties of the ACRES with finer distinction include the possibility to break down the commute route into distinct segments and have the participants to rate each of them. Indeed, further studies in this respect are welcomed.

Possibilities of direct comparisons with previous research regarding validity, are limited by the lack of research in this area and because we study a specific behaviour in a specific environment. Previous studies have mainly focused on physical activity and the neighbourhood. Some validity results have been reported on the Neighborhood Environment Walkability Scale (NEWS) [20-26]. In three studies, one carried out in the USA [26] and two in Australia [23,25], researchers used the NEWS or a modified Australian version (NEWS-AU) in a high- versus low-walkability comparison design. This design is similar to ours in comparing perceptions of two different environments. Our findings are consistent with those of these three studies, supporting the view that differences in environment characteristics can be assessed by self-reports.

### Representativity

The second aim of this study dealt with the fact that active commuters, and particularly bicyclists, normally represent a small group within the general population in larger cities. Therefore, it is, at present, difficult to use population-based random samples. In our previous study, we recruited active commuting participants in the street, reaching for a sufficiently representative group of active commuters. This is, however, a difficult and impractical method when the aim is to reach a large sample of active commuters and to achieve a wide geographical coverage, particularly in larger cities. Therefore, this time we recruited participants by advertising in morning newspapers as an alternative method. Consequently, we were concerned about the representativity of the advertisement-recruited participants and therefore we compared the ratings of the inner urban and suburban route environments between the advertisement- and the street-recruited participants. First, in general, the sex-neutral means, as well as the sex-neutral mean values for the standard deviations, corresponded reasonably well between the advertisement- and the street-recruited participants for both the inner urban and the suburban route environments. Second, overall, the distributions of the response for both the advertisement- and street-recruited men and women ranged from nearly minimum to maximum. This can furthermore be interpreted as support for the use of the 15-point scales, a scale which enables other statistical analyses, for example correlation analyses, and finer distinctions between environments, in contrast to the majority of questionnaires in this area, which have Likert-type scales with fewer response alternatives [e.g. [26]]. Third, correspondence in both the significance and the direction of the differences was noted between the advertisement- and the street-recruited participants in the majority of the items, and for both men and women. Fourth, the sizes of differences between ratings of the inner urban and suburban route environments, expressed as sex-neutral means, generally corresponded reasonably well between the advertisement- and street-recruited participants. Furthermore, although not tested for significance, in general, no major differences appear to exist between the descriptive characteristics of the advertisement- and the street-recruited participants. Thus, overall, there was a good correspondence between the advertisement- and the street-recruited participants. This strengthens the argument for the use of the advertisement-recruited participants in this study as well as recruitment by advertisement as a feasible method. This result is encouraging, since, as stated previously, it is difficult to use population-based random samples, at present, when studying active commuters because they normally only represent a small proportion of the population in larger cities.

### Commuting route environment profiles and bikeability

The third aim of this study involved commuting route environment profiles and the bikeability of different settings. Ratings of suburban route environments were compared to ratings of inner urban route environments for *Both I&S *as well as between *Only I *and *Only S*. In both of these comparisons, we noted significantly higher ratings by both men and women for the suburban environments in traffic safety and the extent to which the route environment stimulated bicycle-commuting. We regard these perceptions as major outcome perceptions with respect to bikeability of route environments (see below). Both of these components of bikeability are most likely composite outcomes of input from several of the environmental predictor items studied with the ACRES. And we view them at this point as potential mediators between the environmental items and the bicycling behaviour.

Given that the route environmental profiles are distinctly different (see Figures 7 and 8), we suggest that the higher ratings of bikeability of route environments in suburban areas are to be explained by factors that differ between these settings. The higher ratings of *bicycle paths *and *greenery *in the suburban as compared to the inner urban route environments, and the lower ratings of *exhaust fumes*, *noise*, *flow of motor vehicles*, *congestion: all types of vehicles*, *congestion: bicyclists*, *conflicts*, *course of the route *and *red lights *are therefore regarded at this point as explanatory candidates for the differences in the outcome perceptions of traffic safety and the extent to which a route environment stimulates or hinders bicycle-commuting. Interestingly, a recent study on motivators and deterrents of bicycling supports many of our findings regarding possible explanatory factors [27].

All our participants are bicyclists and therefore have a 'bicycle use behaviour'. Yet, the environmental conditions vary substantially when they bicycle-commute in the inner urban as compared to the suburban areas. Thus, cycling takes place despite the fact that a route environment is perceived as being, for example, more or less safe. From an analytical perspective, it therefore appears to be useful to differentiate between bicycle usage and the extent to which the route environments are bicycling-friendly, or even hostile.

In the predominant research and scientific dialogue dealing with walkability in more recent years, we most frequently note a paucity of such distinctions [cf. [28,29]]. This could introduce a risk of a rather crude understanding of the linkage between environment and walking behaviour. The research on bikeability is at a much earlier state of development than that of walkability. In the methodological context of this study we therefore consider it to be useful to elaborate on the term bikeability and its definitions.

We suggest that the term bikeability should be used for factors associated with bicycling and the route environment, route distance and aspects of the interaction between the bicyclist and the bicycle which affect the conditions of a specific trip. In our minds, the term bikeability should preferably relate, in a wider perspective, to how these factors and aspects can interact with the perception and behaviour of bicycling for at least three different purposes: (1) transport; (2) recreation; and (3) exercise, as well as competition.

The purpose of bicycling may affect our understanding of bikeability. Given that bicycling with a transport purpose by definition always involves a destination, distinct distance demands are imposed. However, distance-decay relations, i.e. people's willingness to travel different distances to reach destinations [cf. [30]], may vary for different types of destination. Furthermore, the desired qualities of the route environments during cycling with the purpose of recreation and exercise might be higher than for the purposes of transport.

Our focus here will be limited to bikeability in relation to active commuting to one's place of work or study. We suggest that the following different environmental aspects should be included as components of possible importance for the perception of bicycling friendliness in relation to active commuting: (1) the means of transport - the bicycle; (2) the level of safety; (3) whether the route environment stimulates or hinders active commuting; and (4) the route distance and topography.

*The means of transport - the bicycle *- relates to various aspects of the fact that bicycling represents an interaction between a human being and technology, in which the bicycle stands for a technological environment. The effort needed for transport per distance and elevation, the possible speeds and in what kinds of environment it can be used are examples of issues that can affect bikeability. *The level of safety *relates to traffic safety and other forms of risks, such as crime. *Whether the route environment stimulates or hinders active commuting *will most likely relate to a complex of environmental variables (see above). *The route distance and topography *relate to issues of time allocation needed and acceptable levels of physical effort.

These components can be viewed as a chain with four different links. Weakness in one link may be enough to break the chain. However, the characteristics of the different components might also very well interact. For example, a perceived high level of traffic unsafeness may be acceptable if the route distance is sufficiently short. Furthermore, a certain degree of hilliness is not problematic if one has a bicycle with several gears, and so forth. Given this background, we think that it is important to try to study all these aspects of bikeability and the relations between them. We also think that it is important to be specific when using the term bikeability by clearly indicating which aspect of the phenomenon has been studied.

The present results point to a higher level of stimulation of bicycle-commuting and traffic safety in the suburban route environments than in the inner urban ones. We therefore assert that the bikeability of the suburban route environments in these two perspectives is higher than in the inner urban area.

The importance of traffic safety in relation to bikeability is reflected by the fact that the perception of traffic unsafeness is commonly reported as the major hindrance to bicycling [cf. [10]]. It has recently been stated that the likelihood of bicycle use is higher when residential neighbourhood environments have higher residential density, greater mixed land use and higher connectivity of streets [31]. This has been interpreted to be due to, not least, proximity to destination points, which means shorter distances in these areas [31]. If this interpretation is correct, it can, in conjunction with our findings, point in the opposite direction as well: bicycle usage in settings with higher residential density and greater mixed land use may exist under conditions of clearly suboptimal bikeability from a population perspective. Recent findings of higher demands on route environments for transport among occasional and potentially new bicyclists [32] point to the importance of both safe and stimulating route environment qualities from a public health perspective.

Thus, combining proximity to destinations with a high degree of personal safety and stimulating route environments for active transport therefore appears to be an important goal for future urban and regional planning with the aim of creating more widely attractive environmental settings for active commuting. From this perspective, it is important to further our understanding of the bikeability of route environments and the factors that contribute to the perception of traffic safety and whether a route environment stimulates cycling or not. In other words; what constitutes cycling-friendly route environments?

### Limitations

Several possible limitations of the present study should be mentioned. First, the collected data relied on self-reports. More objective measurements may provide additional information about the route environments. On the other hand, studies have shown poor agreement between objective and perceived measures of environments [33-35]. Yet, perceptions of the environments are likely to influence people's physical activity behaviours [cf. [3]]. For example, if people think that the traffic environment is unsafe, although it is in fact safe, their perceptions could result in a non-active commuting behaviour. A combination of objective and perceived measures of the environment may be important to further knowledge about the possible associations between environments and physical activity behaviours. Second, the generalizability of this study is limited. The work with the ACRES is in a relatively early stage at present and we have only assessed it based on one city and two different samples of people. Studies are also desirable regarding active transports with other purposes, different route environments and different samples. Our participants were active commuters and therefore probably very familiar with their route environments. Hence, their perceptions might differ from non-active commuters [36]. Non-active commuters' perceptions of the route environment are important to study to further a more comprehensive knowledge of the route environment in relation to active commuting [cf. [32]]. A slightly modified version of the ACRES could be used for such a purpose. Third, data on the different participant groups was collected during different months as well as during different years: in May, 2005, for the advertisement-recruited participants, in November and December, 2005, for the street-recruited participants and in September and October, 2009, for the expert panel. The compared ratings could therefore be based on somewhat different environments due to, for example, seasonal or built environment changes. On the other hand, only small, if any, changes have occurred in Greater Stockholm during this period, and these changes probably do not have an effect on the general picture of the route environment [cf. [14]]. The street-recruited participants appear to be characterized by all-year round active commuting, whereas the advertisement-recruited participants appear to include summer season active commuting as well. Active commuting is, however, normally a repetitive behaviour along a specific route. As mentioned, this probably makes the active commuters very familiar with their individual route environment and their perceptions of the route environment can therefore be considered relevant, irrespective of rather moderate variations in yearly trip frequency. Fourth, no adjustments for potential confounders were made. The descriptive characteristics of the commuting participants, based on self-reports, yielded a very homogeneous picture, with few distinctions of characteristics allowing for adjustments of potential confounders. The commuting participants were, however, separated into men and women so as to allow useful separate analyses.

### Strengths

Despite the above-mentioned possible limitations, this study has several strengths. One is that we studied active bicycle commuters and their route environments: a specific physical activity behaviour and the specific environment within which the behaviour occurred [cf. [37]]. In accord with this, an important strength of the ACRES is that it deals with the whole commuting route environment and not just the local neighbourhood, as many other questionnaires on physical activity and the environment do [e.g. [26]]. Another strength is that the advertisement-recruited participants provided a large sample size. This enabled, among other things, different groupings, for example, separation of those who bicycle-commuted in both inner urban and suburban areas and those who bicycle-commuted in only an inner urban or a suburban area, for comparisons. Indeed, this design also disclosed a robustness of the ACRES and further strengthened its criterion-related validity. Other important strengths are the criterion-related study design, using an expert panel as a way of handling the problem of non-existing objective data for comparison, and the assessments of representativity, using different samples recruited by different sampling methods. Furthermore, the different commuting route environment profiles of inner urban and suburban areas provide a basis for furthering the understanding of bikeability in relation to route environments. To our knowledge, this is a new approach.

## Conclusions

In conclusion, the overall results show (a) considerable criterion-related validity of the ACRES, (b) ratings of advertisement-recruited participants mirroring those of street-recruited participants and (c) different commuting route environment profiles of inner urban and suburban areas. Suburban environments were rated as safer and more stimulating for bicycle-commuting than the inner urban environments, demonstrating a greater bikeability of route environments in suburban areas. Consequently, the results support the use of the ACRES in future research to assess bicyclists' perceptions of their route environments, as well as by health and transport professionals to survey route environments for other purposes.

## Competing interests

The authors declare that they have no competing interests.

## Authors' contributions

PS and LW contributed to the design of the study. PS was involved in the data acquisition regarding the advertisement- and street-recruited participants, as well as the expert panel. LW was responsible for data acquisition regarding the expert panel, checked the data from the PACS Q2 for accuracy, performed the statistical analyses and drafted the first version of the manuscript. PS drafted the manuscript and supervised LW as part of her PhD training. Both authors read and approved the final manuscript.

## Pre-publication history

The pre-publication history for this paper can be accessed here:

http://www.biomedcentral.com/1471-2288/11/6/prepub

## References

[B1] World Health Organization (WHO) Global Strategy on Diet, Physical Activity, and Health 2004 Geneva: WHO

[B2] Shephard RJ Is active commuting the answer to population health? Sports Med 2008 38 9 751 758 10.2165/00007256-200838090-00004 18712942

[B3] Sallis JF Cervero RB Ascher W Henderson KA Kraft MK Kerr J An ecological approach to creating active living communities Annu Rev Public Health 2006 27 297 322 10.1146/annurev.publhealth.27.021405.102100 16533119

[B4] Gebel K Bauman AE Petticrew M The physical environment and physical activity: a critical appraisal of review articles Am J Prev Med 2007 32 5 361 369 10.1016/j.amepre.2007.01.020 17478260

[B5] Brownson RC Hoehner CM Day K Forsyth A Sallis JF Measuring the built environment for physical activity: state of the science Am J Prev Med 2009 36 Suppl 4 99 123 10.1016/j.amepre.2009.01.005 PMC2844244 19285216

[B6] Alexander A Bergman P Hagströmer M Sjöström M IPAQ environmental module; reliability testing J Public Health 2006 14 76 80 10.1007/s10389-005-0016-2

[B7] Hu G Pekkarinen H Hänninen O Yu Z Tian H Guo Z Nissinen A Physical activity during leisure and commuting in Tianjin, China Bull World Health Organisation 2002 80 933 938 PMC2567698 12571720

[B8] Stigell E Schantz P Physically active commuting between home and work/study place in Greater Stockholm [abstract] Proceedings from Transport Research Arena Europe. Greener, safer and smarter road transport for Europe. Conference of European Directors of Roads, European Commission & European Road Transport Research Advisory Council: 12-15 June 2006; Göteborg, Sweden 2006 109

[B9] Wahlgren L Stigell E Schantz P The active commuting route environment scale (ACRES): development and evaluation Int J Behav Nutr Phys Act 2010 7 58 10.1186/1479-5868-7-58 20609250 PMC2914072

[B10] Parkin J Ryley T Jones T Horton D, Rosen P, Cox P Barriers to cycling: an exploration of quantitative analyses Cycling and Society 2007 Aldershot, UK: Ashgate 67 82

[B11] Cronbach J Meehl P Construct validity and psychological tests Psychological Bulletin 1955 52 4 281 302 10.1037/h0040957 13245896

[B12] The Stockholm - Uppsala Air Quality Management Association Stockholm - Uppsala Air Quality Management Association [web site] 2009 Swedish: Stockholm och Uppsala Läns Luftvårdsförbund http://www.slb.nu/lvf/

[B13] The Municipality of Stockholm, Sweden, The Environment Department The Noise Map of Stockholm 2009 Swedish: Stockholms Stad, Miljöförvaltningen: Stockholms bullerkarta http://www.map.stockholm.se/kartago/kartago_fr_buller.html

[B14] Eliasson J Lessons from the Stockholm congestion charging trial Transport Policy 2008 15 395 404 10.1016/j.tranpol.2008.12.004

[B15] Morán Toledo CA Framework for estimating congestion performance measure: from data collection to reliability analysis: case study of Stockholm Licentiate thesis 2008 Royal Institute of Technology Stockholm, Sweden, Department of Transport and Economics

[B16] The Stockholm Trial Stockholm Trial [web site] 2009 Swedish: Stockholmsförsöket http://www.stockholmsforsoket.se

[B17] Löfvenhaft K Spatial and temporal perspectives on biodiversity for physical planning: examples from urban Stockholm, Sweden PhD thesis 2002 Stockholm University, Sweden, The Department of Physical Geography and Quaternary Geology

[B18] The Municipality of Stockholm, Sweden, The Research and Statistics Office Area and population density by City district 2008-12-31 Swedish: Stockholms Stad, Utrednings- och Statistikkontoret: Areal och befolkningstäthet i stadsdelsområden, SDN-delar och stadsdelar http://www.usk.stockholm.se/arsbok/b039.htm

[B19] Turner T Niemeier D Travel to work and household responsibility: new evidence Transportation 1997 24 397 419 10.1023/A:1004945903696

[B20] Adams MA Ryan S Kerr J Sallis JF Patrick K Frank LD Norman GJ Validation of the Neighborhood Environment Walkability Scale (NEWS) items using Geographic Information Systems J Phys Act Health 2009 6 Suppl 1 113 123 10.1123/jpah.6.s1.s113 19998857

[B21] De Bourdeaudhuij I Sallis JF Saelens BE Environmental correlates of physical activity in a sample of Belgian adults Am J Health Promot 2003 18 1 83 92 13677966 10.4278/0890-1171-18.1.83

[B22] Cerin E Saelens BE Sallis JF Frank LD Neighborhood Environment Walkability Scale: validity and development of a short form Med Sci Sports Exerc 2006 38 9 1682 1691 10.1249/01.mss.0000227639.83607.4d 16960531

[B23] Cerin E Leslie E Owen N Bauman A An Australian version of the Neighborhood Environment Walkability Scale: validity evidence Meas Phys Educ Exerc Sci 2008 12 31 51 10.1080/10913670701715190

[B24] Cerin E Conway TL Saelens BE Frank LD Sallis JF Cross-validation of the factorial structure of the Neighborhood Environment Walkability Scale (NEWS) and its abbreviated form (NEWS-A) Int J Behav Nutr Phys Act 2009 6 32 10.1186/1479-5868-6-32 19508724 PMC2700069

[B25] Leslie E Saelens B Frank L Owen N Bauman A Coffee N Hugo G Residents' perceptions of walkability attributes in objectively different neighborhoods: a pilot study Health Place 2005 11 227 236 10.1016/j.healthplace.2004.05.005 15774329

[B26] Saelens BE Sallis JF Black JB Chen D Neighborhood-based differences in physical activity: an environment scale evaluation Am J Public Health 2003 93 1552 1558 10.2105/AJPH.93.9.1552 12948979 PMC1448009

[B27] Winters M Davidson G Kao D Teschke K Motivators and deterrents of bicycling: comparing influences on decisions to ride Transportation 2011 38 1 153 168 10.1007/s11116-010-9284-y

[B28] Frank LD Schmid TL Sallis JF Chapman J Saelens BE Linking objectively measured physical activity with objectively measured urban forms: findings from SMARTRAQ Am J Prev Med 2005 28 2S2 117 125 10.1016/j.amepre.2004.11.001 15694519

[B29] Inoue S Ohya Y Odagiri Y Takamiya T Ishii K Kitabayashi M Suijo K Sallis JF Shimomitsu T Association between perceived neighborhood environment and walking among adults in 4 Cities in Japan J Epidemiol 2010 20 4 277 286 10.2188/jea.JE20090120 20472982 PMC3900787

[B30] Iacono M Krizek K El-Geneidy A Access to destinations: how close is close enough? Estimating accurate distance decay functions for multiple modes and different purposes Access to destinations study 2008 Minnesota Department of Transportation Report # 4 in the series

[B31] Owen N De Bourdeaudhuij I Sugiyama T Leslie E Cerin E Van Dyck D Bauman A Bicycle use for transport in an Australian and a Belgian city: associations with built-environment attributes Journal of Urban Health: Bulletin of the New York Academy of Medicine 2010 87 2 189 198 20174879 10.1007/s11524-009-9424-x PMC2845830

[B32] Winters M Teschke K Route preferences among adults in the near market for bicycling: findings of the Cycling in Cities study Am J Health Promot 2010 25 1 40 47 10.4278/ajhp.081006-QUAN-236 20809831

[B33] Ball K Jeffery RW Crawford DA Roberts RJ Salmon J Timperio AF Mismatch between perceived and objective measures of physical activity environments Preventive Medicine 2008 47 294 298 10.1016/j.ypmed.2008.05.001 18544463

[B34] Hoehner CM Brennan Ramirez LK Elliott MB Handy SL Brownson RC Perceived and objective environmental measures and physical activity among urban adults Am J Prev Med 2005 28 2S2 105 116 10.1016/j.amepre.2004.10.023 15694518

[B35] McGinn AP Evenson KR Herring AH Huston SL Rodriguez DA Exploring associations between physical activity and perceived and objective measures of the built environment Journal of Urban Health: Bulletin of the New York Academy of Medicine 2007 84 2 162 184 17273926 10.1007/s11524-006-9136-4 PMC2231636

[B36] Titze S Stronegger WJ Janschitz S Oja P Environmental, social, and personal correlates of cycling for transportation in a student population J Phys Act Health 2007 4 66 79 17489008 10.1123/jpah.4.1.66

[B37] Giles-Corti B Timperio A Bull F Pikora T Understanding physical activity environmental correlates: increased specificity for ecological models Exerc Sport Sci Rev 2005 33 4 175 181 10.1097/00003677-200510000-00005 16239834

